# Pragmatic and executive functions in traumatic brain injury and right brain damage: An exploratory comparative study

**DOI:** 10.1590/S1980-57642011DN05040013

**Published:** 2011

**Authors:** Nicolle Zimmermann, Gigiane Gindri, Camila Rosa de Oliveira, Rochele Paz Fonseca

**Affiliations:** 1 Clinical and Experimental Neuropsychology Research Group, Post-graduate Program in Psychology (Human Cognition), Pontifical Catholic University of Rio Grande do Sul (PUCRS), Porto Alegre RS, Brazil; 2 Master’s Student at PUCRS, supported by the Conselho Nacional de Desenvolvimento Científico e Tecnológico (CNPq); 3 PhD Student at PUCRS, supported by Coordenação de Aperfeiçoamento Pessoal de Nível Superior (CAPES)

**Keywords:** brain injuries, stroke, executive function, communication disorders

## Abstract

**Objective:**

To describe the frequency of pragmatic and executive deficits in right brain damaged (RBD) and in traumatic brain injury (TBI) patients, and to verify possible dissociations between pragmatic and executive functions in these two groups.

**Methods:**

The sample comprised 7 cases of TBI and 7 cases of RBD. All participants were assessed by means of tasks from the Montreal Communication Evaluation Battery and executive functions tests including the Trail Making Test, Hayling Test, Wisconsin Card Sorting Test, semantic and phonemic verbal fluency tasks, and working memory tasks from the Brazilian Brief Neuropsychological Assessment Battery NEUPSILIN. Z-score was calculated and a descriptive analysis of frequency of deficits (Z< -1.5) was carried out.

**Results:**

RBD patients presented with deficits predominantly on conversational and narrative discursive tasks, while TBI patients showed a wider spread pattern of pragmatic deficits. Regarding EF, RBD deficits included predominantly working memory and verbal initiation impairment. On the other hand, TBI individuals again exhibited a general profile of executive dysfunction, affecting mainly working memory, initiation, inhibition, planning and switching. Pragmatic and executive deficits were generally associated upon comparisons of RBD patients and TBI cases, except for two simple dissociations: two post-TBI cases showed executive deficits in the absence of pragmatic deficits.

**Discussion:**

Pragmatic and executive deficits can be very frequent following TBI or vascular RBD. There seems to be an association between these abilities, indicating that although they can co-occur, a cause-consequence relationship cannot be the only hypothesis.

## Abstract (pt)

**Objetivo:**

Descrever a frequência de déficits pragmáticos e executivos em pacientes com lesão de hemisfério direito (LHD) e com traumatismo cranioencefálico (TCE), e verificar se existem dissociações entre pragmática e funções executivas nesses dois grupos.

**Métodos:**

A amostra foi composta de 7 casos de TCE e 7 casos de LHD. Todos os participantes foram avaliados por tarefas da Bateria Montreal de Avaliação da Comunicação e com testes de funções executivas como o Trail Making Test, Teste Hayling, Wisconsin Card Sorting Test, tarefas de fluência verbal semântica, fonêmica e livre, tarefas de memória de trabalho do Instrumento de Avaliação Neuropsicológica Breve NEUPSILIN. O escore Z foi calculado e a freqüência de déficits (Z< -1.5) foi descrita.

**Resultados:**

Os pacientes com LHD apresentaram déficits principalmente no discurso conversacional e narrativo, por enquanto que os pacientes com TCE demonstraram um padrão mais difuso de déficits pragmáticos. Quanto as FE, os déficits dos pacientes com LHD foram de memória de trabalho e iniciativa verbal, principalmente. Por outro lado, os pacientes com TCE apresentaram novamente um padrão mais geral de disfunção executiva, com prejuízos principalmente de memória de trabalho, iniciativa, inibição, planejamento e switching. Em geral, os déficits pragmáticos e executivos foram associados comparando os pacientes com LHD e com TCE, exceto por duas dissociações simples: dois casos de TCE demonstraram déficits executivos com ausência de déficits pragmáticos.

**Discussão:**

Os déficits pragmáticos e executivos podem ser muito frequentes após um TCE ou uma LHD. Parece haver uma associação entre essas habilidades, ressaltando-se que apesar de elas poderem co-ocorrer, uma relação de causa-consequência não parece ser a única hipótese.

Pragmatic deficits are among the most frequent and relevant impairments in communication after acquired brain damage, particularly in right brain damage (RBD) and traumatic brain injury (TBI).^1,2^ Several studies have shown clinical evidence of difficulties among these patients to communicate in an appropriate and effective way in interaction contexts,^3^ with various similarities among cases.^4^ Such deficits lead to the inability to understand non-literal meanings (metaphors), indirect language, jokes or ironies during conversation. In addition, patients can fail to respect their turn to talk or to maintain eye contact with the interlocutor.^5-7^ In general, pragmatic abilities are related to communicative intention^8^ as well as to the usage of language in context.^9^

For many years, studies investigated pragmatics as an essentially linguistic ability with little emphasis on other cognitive functions that might contribute to successful communication. Nonetheless, advances in the field of cognitive neuroscience have led to new approaches to understanding pragmatic deficits, such as the hypothesis of EF (EF) influence on these communication abilities.^10^ This relationship was originally derived from studies on TBI, due to the tendency of this population to present frontal damage. With regards to RBD patients, McDonald^11^ suggested that executive functioning would not account for pragmatic deficits in this sample. The author investigated a RBD sample, conducting a correlation analysis between pragmatic and cognitive functions, from which he found positive correlations between pragmatic and visuospatial components but not between pragmatic and EF tasks.

In light of these findings, there is no consensus regarding the role of EF in the pragmatic impairment of these two populations, while similarities or differences between RBD and TBI samples remain unclear. Regarding the main focus of clinical neuropsychological assessment, namely, the comprehension of cognitive dissociations and associations, and the study of the hypothesis of secondary pragmatic deficits due to primary executive dysfunction, remain an open challenge. Therefore, the aim of this paper was to present an exploratory study on the frequency of pragmatic and executive deficits post-RBD and TBI, as well as to verify possible dissociations between these functions in the two groups.

## Methods

### Participants

Seven adults with stroke in the right hemisphere and seven adults with TBI were recruited from outpatient records of hospitals in Porto Alegre, Brazil. All participants were Brazilian Portuguese native speakers and were aged from 18 to 55 years old, and had an education level ranging from 3 to 18 years. Subjects were evaluated at least one month post-onset. None of the patients presented moderate to severe aphasia assessed by oral and written language tasks from the Brazilian Brief Neuropsychological Assessment Battery NEUPSILIN.^12,13^ The RBD participants had no history of other neurological or psychiatric disorders and had suffered a single vascular unilateral brain lesion. No history of substance abuse (such as alcohol, tobacco or illicit drugs) was reported in the RBD group. The TBI group, had no history of other neurological disorders, but three cases had reported history of psychiatric treatment and substance abuse. These disorders frequently co-occur, where TBI is more prevalent in adults with alcohol and other drugs abuse and other psychiatric disorders.^14-17^ Characteristics of the brain lesion were obtained by neuroimaging and routine neurological exams. Sociodemographic and clinical characteristics of the two groups are described in detail by group in Table 1. All participants signed an Informed Consent Form.

**Table 1:** Sociodemographic and clinical characterization of TBI and RBD participants.

Participants	Age*	Education*	Sex	MMSE^+^	Type of lesion	Lesion site	Time post- onset (months)
RHD 1	34	17	F	29	CVAi	R perinsular	41
RHD 2	40	3	M	21	CVAi	R subventricular	11
RHD 3	41	5	F	26	CVAi	R frontal+parietal	2
RHD 4	46	4	F	26	CVAh	R parietal+temporal	15
RHD 5	47	7	F	22	CVAi	R base nuclei + R periventricular	63
RHD 6	49	9	F	24	CVAi	R base nuclei + R cerebral artery	1
RHD 7	32	18	F	29	CVAi	R parietal	31
TBI 1	18	11	M	**	Severe TBI	R frontal lobe + L cerebellar + L temporo-parieto-occipital transition	7
TBI 2	23	10	F	**	Severe TBI	L temporal pole	44
TBI 3	28	14	F	**	Severe TBI	L temporal	11
TBI 4	32	12	M	**	Severe TBI	R temporal + diffuse hemorrhage	90
TBI 5	33	13	F	**	Mild TBI	R temporal + R parietal + diffuse axonal injury	9
TBI 6	35	14	M	**	++	L frontal + L temporal	3
TBI 7	55	11	M	30	++	R temporal	11

R: right; L: left; CVAi: cerebrovascular accident - ischemic; CVAh: cerebrovascular accident - hemorrhagic;

+ Mini Mental State Examination;

* in years;

** only assessed in individuals aged 40 years or older. ++Missing data.

++ Missing data.

### Instruments

All participants were assessed by means of a battery of EF tasks and some pragmatic-inferential subtests from the Montreal Communication Evaluation Battery,^18,19^ an instrument adapted to Brazilian Portuguese.^20^ The administered tools are described below.

#### PRAGMATIC PROCESSING ASSESSMENT

##### Montreal Communication Evaluation Battery^18^

***Conversational discourse*** - In this task, a 10-minute conversation is recorded between the examiner and participant about a familiar topic. Some criteria are used to evaluate pragmatics: level of search or change of words, occurrence of imprecise ideas, inappropriate or unintelligible comments, change of subject, verbal initiative, among others. The maximum score is 34.

***Metaphor interpretation*** - Metaphoric sentences are read to the participants and must attempt to explain them, total score /40.

***Narrative discourse*** - In the partial narrative discourse part, short paragraphs are read to the participants, which they are expected to recount after each is read. Subsequently, subjects listen to the full text without intervals and then have to recount the whole story. Some questions about the text, that require inferential abilities, are then posed. Scoring is divided into essential information recalled (maximum 18 points), present information recalled (maximum 29 points), integral narrative discourse recalled (maximum 13 points) and comprehension questions (maximum 12 points)

***Indirect speech acts interpretation*** - The examiner reads to the participants some stories that have direct and indirect speech acts. The task requires participants to judge the intentions of the characters from the story. The maximum score is 40.

##### EF assessment

***Trail Making Test***^21,22^ - This task assesses processing speed, inhibition and cognitive flexibility. It consists of a paper and pencil instrument, in which the participant must connect numbers (Part A) and alternate between numbers and letters (Part B) in a specific order. It is divided into Parts A and B on which time and accuracy scores are recorded. The maximum accuracy score is 24 and time of execution 300 seconds.

***Hayling Test***^23,24^ - Verbal initiation and inhibition and planning are the main functions evaluated by this task. Participants must complete orally a sequence of sentences in which the last word is missing. This task comprises two parts: A (automatic responses) and B (response suppression). In both, time taken to access the word and number of correct answers (maximum of 15) are analyzed. Specifically for Part B, a quantitative score from qualitative analysis is also provided (maximum 45 points).

***Wisconsin Card Sorting Test***^22,25^ - This evaluates executive components such as planning, abstraction, maintenance of successful strategies and cognitive flexibility and was chosen for its specific design for brain damaged patients. The goal of the task is to match or associate geometric pictures in accordance to rules that the participant must find out. Number of completed categories (maximum of 6), perseverative errors (maximum of 46), non-perseverative errors (maximum of 47) and ruptures (maximum of 12) were analyzed.

***Montreal Communication Evaluation Battery***^18^ - Phonemic, semantic and unconstrained verbal fluency tasks - These tasks evaluate verbal planning, initiation and inhibition, including lexical search strategies. The phonemic-orthographic criterion (2 minutes) was the letter "P", the semantic criterion (2 minutes) was "clothes" and the unconstrained task (2 minutes and 30 seconds) had no criterion.

##### Tasks from the Brazilian Brief Neuropsychological Assessment Battery NEUPSILIN^12,13^

***Ascendant ordering of digits*** - Participants listen to a series scrambled numbers and must organize them in an ascendant order. It mainly evaluates the central executive and phonological loop components of working memory. One point was assigned for each correct answer, giving a total score of 10 and a maximum qualitative span score of six.

***Oral word span in sentences*** - In this task, sentences are read to participants who must record the last word of each phrase and recall them in order at the end of each trial. The executive component evaluated is again the central executive of working memory. Maximum total score is 28 and the maximum qualitative score for set of words repeated correctly is five.

***Problem solving*** - This task consists of two simple problem solving questions, with each question scoring one point. The examiner reads the questions and the participant answers them orally.

### Data analysis

In order to compare different task scores, a Z score was individually calculated based on the normative data of each instrument, which can be analysed in detail using the Brazilian references cited in Instrument sessions. Z score is generally used in order to reduce the possible impact of age and schooling on cognitive performance. Moreover, this procedure allows comparison among tasks with different score scales. As established in previous studies, impaired performance was considered for a Z score cut-off of -1.5 SD. This analysis and its respective cut-off score is frequently used for data interpretation in TBI^26^ and RBD patients.^27^ A correlational analysis was also conducted in the whole sample in order to verify any strong relationships between pragmatic and executive performance in both groups.

## Results

Results of the percentage of deficits for the two groups on the pragmatic and EF tasks are shown in Table 2.

**Table 2:** Performance of RBD and TBI groups on EF and pragmatic tasks.

	RBD 1	RBD 2	RBD 3	RBD 4	RBD 5	RBD 6	RBD 7	% of deficits	TBI 1	TBI 2	TBI 3	TBI 4	TBI 5	TBI 6	TBI 7	% of deficits
Pragmatic tasks / Participants																
Conversational discourse	-3.42	-4.74	-4.83	-5.4	0.85	0.28	-2.6	71%	-9.46	-3.42	-5.4	-11.3	-1.45	2.8	-0.97	57%
Metaphor	0.84	0.53	-1.32	-0.28	0.94	-1.15	-0.05	0	-0.7	-0.7	-4.08	-4.39	0.11	-0.89	-0.3	29%
Partial narrative discourse - essential information	-1.64	-0.4	-0.43	-0.74	0.49	-1.97	-3.72	43%	-2.04	-2.05	-2.05	0.08	-0.4	-2.04	-1.31	57%
Partial narrative discourse - present information	-0.83	1.18	0.61	0.84	0.84	-2.11	-2.48	29%	-1.69	-1.7	-1.98	-0.83	-1.4	-2.84	-1.37	57%
Integral narrative discourse	-0.82	0.08	-0.81	-1.55	0.67	-2.29	-2.17	43%	-2.47	-1.72	0.82	-2.17	0.01	-1.48	0.32	43%
Narrative discourse - questions	0.13	0.75	-0.66	-1.00	0.68	0.34	0.84	0%	0.13	0.13	-0.47	-4.12	0.13	-0.48	-0.38	14%
Indirect speech acts	-1.7	0.01	-0.7	-0.2	1.29	-0.45	-1.28	14%	0.6	-1.01	-2.38	-4.09	-0.37	-2.31	0.69	43%
EF tasks / Participants																
TMT A time	0.79	-0.52	-0.45	-0.86	-1.32	-1.02	-24.36	14%	-1.35	-17.36	-0.5	-2.32	-0.18	-0.38	0.7	14%
TMT A errors	-0.26	-0.26	-0.27	3.58	-0.27	-0.27	17.95	0%	-0.26	-0.26	-0.26	-0.26	-0.26	-0.26	-0.23	0%
TMT B time	0.8	-0.76	-1.19	-1.21	-1.21	-1.21	-5.57	14%	0.69	*	*	-3.46	0.42	0.42	-0.67	14%
TMT B errors	-0.58	-0.58	0.5	2.91	0.5	-0.51	4.05	0%	-0.58	-0.58	-0.58	0.74	-0.58	-0.58	-0.95	0%
TMT B-A	-0.55	0.61	1.22	1.14	1.02	1.1	-1.56	14%	0.18	-0.14	0.68	2.8	-0.54	-0.72	0.99	0%
TMT B/A	-0.26	0.12	0.9	0.53	0.15	0.38	-1.54	14%	-0.4	-1.08	0.19	0.56	-0.62	-0.82	1.54	0%
TMT B-A/A	-0.26	0.12	0.9	0.53	0.15	0.38	-1.54	14%	-0.4	-1.08	0.19	0.56	-0.62	-0.82	1.54	0%
WCST categories	0.59	0.59	-1.16	0	1.16	-0.58	-2.06	14%	0.59	-0.09	-2.79	-0.09	-0.09	0.59	0.86	14%
WCST perseverative errors	-0.59	-0.75	2.33	0.95	-0.57	2.74	2.95	0%	-0.75	0.69	-0.91	-0.43	0.21	-0.43	-0.61	0%
WCST non-perseverative errors	-0.58	-0.84	5.8	4.57	1.18	8.57	7.08	0%	-0.33	-0.07	-0.58	0.18	-0.33	-0.33	0.31	0%
WCST ruptures	0.71	0.71	6.45	0.84	0.84	0.84	8.21	0%	-0.58	-0.58	0.71	0.71	0.71	0.71	-0.56	0%
Hayling A time	0.94	0.68	-1.98	-0.92	-0.64	-6.11	-13.11	43%	-1.19	-1.98	-0.44	-3.1	1.17	-6.34	-0.84	29%
Hayling A errors	1.73	-0.49	2.69	1	-0.69	-0.69	2.05	0%	-0.49	-0.49	1.73	-0.49	-0.49	1.73	-0.45	0%
Hayling B time	1.09	0.63	-1.28	0.08	-5.72	-9.34	-0.83	0%	0.51	0.56	0.43	-0.14	1.06	-1.86	-1.18	14%
Hayling B quantitative errors	-1.21	1.88	1.27	1.78	-0.24	1.02	1.64	0%	-0.28	-0.59	-1.21	-0.9	-0.59	0.65	-1.34	0%
Hayling B qualitative errors	-1.18	*	1.24	2.34	-0.69	0.87	2.49	0%	-0.29	-0.41	-1.18	-0.79	-0.67	0.35	-1.44	0%
Hayling B-A	-0.98	-0.52	0.81	-0.29	5.24	7.74	-2.29	0%	-1.04	-1.38	-0.68	-0.93	-0.86	-0.01	0.96	0%
Unconstrained verbal fluency	0.42	1.76	-1.43	-1.22	0.89	-1.16	-1.74	14%	-1.06	-1.75	0.62	-1.92	1.21	-1.69	-0.2	43%
Orthographic verbal fluency	-0.01	1.73	-1.58	0.22	0.63	-1.16	-0.9	14%	-2.51	*	-1.74	-2.6	-0.95	-1.89	-0.15	50%
Semantic verbal fluency	-0.47	0.96	-1.55	-1.55	0.57	-0.84	-2.27	43%	-2.79	*	-2.22	-3.01	-0.35	-3.18	-0.05	67%
Ascendant digit longest span	-1.35	-1.35	-0.41	-3.38	-0.02	-1.81	-1.62	43%	-1.95	-2.56	-1.35	-3.16	-1.35	-1.95	-1.01	43%
Ascendant digit span total score	2.96	4.12	0.35	-4.1	0.9	-0.62	0.82	0%	1.79	-0.54	2.96	-2.86	4.12	0.63	5.05	0%
Word-phrase span longest span	-3.06	-2.88	-1.84	-2.36	-1.25	-2.79	-3.2	86%	-2.88	-3.06	-2.88	-3.42	-2.88	-2.88	-3.2	100%
Word-phrase span total score	9.28	11.89	2.09	4.97	10.46	0.65	12.73	0%	8.41	5.8	8.41	4.05	10.15	9.28	10.92	0%
Problem solving	0.38	0.38	-2.3	0.54	-0.72	0.54	-2.65	14%	-2.59	*	-2.59	0.38	0.38	0.38	0.37	33%

Percentage was calculated by the incidence of Z score ≤ -1.5. Deficit was considered when Z score ≤ -1.5.

* missing value; TMT: Trail Making Test; WCST: Wisconsin Card Sorting Test.

As can be seen in Table 2, the comparison between TBI and RBD patients showed that the former cases apparently had more deficits in pragmatic processing than did the RBD group, except for Conversational Discourse and on integral recall of information in the Narrative Discourse task. Percentage of deficits on EF tasks also demonstrated a tendency of TBI individuals to have more deficits than RBD subjects.

Regarding dissociations between pragmatics and EF, these were confirmed when at least one pragmatic or executive variable was impaired. No dissociations were found in RBD cases. In the TBI group however, two cases showed pragmatic-executive dissociation.

Results of correlational analysis based on Spearman coefficients of each performance variable are given in Table 3. This analysis encompassed the whole sample and indicated that metaphors were strongly related to processing speed, lexical-semantic access, switching, and working memory. Moreover, the essential information of the narrative discourse were found to be associated with processing speed, flexibility and inhibition, while the present information was associated only with processing speed. Integral narrative discourse seemed to be related to inhibition, processing speed, planning, lexical-semantic access, flexibility and working memory. The questions about the narrative story were related to working memory abilities only. Finally, indirect speech acts were shown to be related to lexical-semantic access, flexibility and working memory.

**Table 3:** Correlational analysis between pragmatic and executive function tasks in whole sample.

Tasks/analysis	Trail Making Test								Wisconsin Card Sorting Test					Hayling Test							Verbal fluency				Working memory tasks				Problem Solving
	**Time** ? **A**	**Errors** **A**	**Time** **B**	**Errors** **B**	**B-A**	**B/A**	**B-A/A**		**Cat**	**P** **Errors**	**NP** **Errors**	**Rup**		**Time** **A**	**Errors** **A**	**Time** **B**	**Errors** **B/15**	**Errors** **B/45**	**B-A**		**UVF**	**PVF**	**SVF**		**AD** **span**	**AD** **total**	**OW** **span**	**OW** **total**	
Conversational discourse																													
Pearson																													
Sig																													
Metaphor																													
Pearson			-0.643		-0.664																0.623	0.718	0.629				0.738	0.820	
Sig			0.024		0.010																0.017	0.006	0.021				0.003	0.000	
Partial narrative discourse - essential information																													
Pearson		-0.624							0.660	-0.785	-0.821			-0.693				-0.630											
Sig		0.017							0.010	0.001	0.000			0.006				0.021											
Partial narrative discourse - present information																													
Pearson														-0.757															
Sig														0.002															
Integral narrative discourse																													
Pearson			-0.680							-0.701	-0.745			-0.627			-0.604	-0.772			0.780	0.653	0.732		0.793	0.815	0.670	0.798	
Sig			0.015							0.005	0.002			0.016			0.022	0.002			0.001	0.016	0.004		0.001	0.000	0.009	0.001	
Narrative discourse - questions																													
Pearson																									0.704	0.686	0.675	0.645	
Sig																									0.005	0.007	0.008	0.013	
Indirect speech acts																													
Pearson																							0.636				0.631		
Sig																							0.020				0.016		

Cat: categories; P: perseverative; NP: non-perseverative; Rup: ruptures; UVF: unconstrained verbal fluency; PVF: phonemic verbal fluency; SVF: semantic verbal fluency; AD: ascendant ordering of digits; OW: oral word span in sentences.

## Discussion

The aim of this study was to present preliminary data on the frequency of pragmatic and executive deficits in RBD and TBI patients and to discuss the associations or dissociations found between these abilities upon comparing the clinical groups. TBI patients had a greater percentage of deficit on all pragmatic tasks except conversational discourse and narrative discourse. Similarly, TBI cases showed a higher prevalence of executive deficits. The correlational analysis demonstrated that in the sample as a whole, pragmatic and executive functions were strongly related.

### Pragmatic processing in RBD and TBI

It is well established that approximately 50% to 78% of RBD patients exhibit communicative impairment on one or more components.^28^ The subgroup of patients presenting deficits has been further investigated for communicative sub-profiles. Results of the performance in RBD were similar to the cluster or profile described as predominantly discoursive.^27,29^ The difficulties of these individuals producing accurate discourse seem to stem from impaired ability to deal with the macrostructure of discourse, such as topic organization and coherence, as well as inference and figurative and non-literal language processing.^30^ In addition, some authors suggest that discursive impairment might be associated with the ability to maintain and/or suppress information.^31^ Deficits in non-literal comprehension also appear to impact performance on narrative discourse tasks. These patients might have difficulties grasping the context of stories^3^ and understanding the moral of the story.^1^

Results obtained in the pragmatic assessment of TBI patients have demonstrated that this group presents a diffuse pattern of deficits. Recent studies corroborate these results, such as impairment in comprehending and producing indirect speech acts; emotional prosody; and a tendency to show perseveration in conversational discourse.^9^

More specifically, discourse impairment is illustrated by difficulties answering open questions, organizing discourse, understanding and adapting to interlocutor knowledge, introducing new topics and using prosody.^32^ Impairment in discriminating direct and indirect speech acts is also corroborated.^33^ Regarding the comprehension questions about the narrative discourse task, the divergent results found between the present study and the work by Ferstl, Walther, Guthke and von Cramon^34^ highlights two hypotheses:

(1) summing up 12 questions in a single score can reduce the sensitivity of this task for the diagnosis of linguistic-mnemonic deficits;
(2) 43% of both clinical groups presented narrative recounting deficits while only one TBI patient showed deficits answering the comprehension questions.

These samples may be compensating a mnemonic impairment by taking advantage of this recognition task. Metaphor processing is also a frequent impairment, especially in novel metaphors. Metaphorical processing deficits have been linked to difficulties in semantic memory and to compromised connection between frontal and other brain regions.^35^

### Executive functions in RBD and TBI

There appears to be no special or focused interest in the role of the right hemisphere in EF processing. Its role in these complex abilities has been derived from exploratory theories about underlying mechanisms of communicative impairments in RBD patients^33,36,37^ and from neuroimaging studies investigating brain regions associated to EF components.^38^ Results presented in this article suggest verbal working memory and initiation deficits in RBD patients. Gindri, Zibetti and Fonseca^39^ found similar results using the Hayling test in a larger sample of RBD stroke patients, but contradictory findings were described by Champagne-Lavau and Joanette^40^ in which authors found difficulties on the inhibition section of the test (part B). These results are discordant probably due to the different communication profiles of the respective samples. In the present study, RBD patients presented mainly discursive dysfunction while in Champagne-Lavau and Joanette's study, problems were characterized by pragmatic impairment. In as far as the main hypothesis of the relationship between pragmatic and executive deficits is based on performance in non-literal sentences, the most frequent working memory deficits found in the present study can be explained by the greater complexity of the discursive stimuli used in the communication assessment. Thus, pragmatic deficits after RBD cannot be associated only to inhibitory deficits but also to working memory dysfunction.

Regarding executive dysfunction in TBI individuals, this patient group proved to be predominantly impaired in verbal working memory, verbal initiation, inhibition, planning and switching (verbal fluency tasks) components. Interestingly, in spite of the well documented executive dysfunction observed in TBI patients, the cases included in this sample did not present impaired performance on any measures of EF.

Regarding the perspective of working memory function, a similar result was found in the study by Anderson and Knight,^42^ in which the TBI group differed in performance from normal controls on a central executive task and in social functioning scales, but did not differ on other EF measures (TMT, Controlled Oral Word Association Task, WCST). This finding probably demonstrates the limitations of standardized non-ecological measures of EF assessment in this population. Concerning the findings from the verbal fluency task, our results demonstrated that the semantic verbal fluency task is the most affected measure in the TBI group, in line with findings of other investigations,^26^ followed by the orthographic and unconstrained task, respectively. Although a qualitative analysis of the tasks was not included in this study, previous studies have put forward the hypothesis that this phenomenon might be due to difficulties in switching between subcategories.^26^

### Dissociations between pragmatics and EF abilities in RBD and TBI

There seems to be an association between pragmatic deficits and the EF component central executive of working memory, verbal planning, initiation and inhibition, switching, shifting and strategy maintenance related to verbal fluency.^43^ Similar results were found in a group study with TBI patients^44^ in which moderate correlations were found between factors of a pragmatic instrument and phonemic verbal fluency tasks. This study highlighted that one third of the pragmatic difficulties in TBI patients might be due to EF problems. The strong correlations obtained in the second analysis emphasized these associations.

Besides the main findings regarding pragmatics and EF in TBI and RBD, the most prevalent deficits in discursive tasks compared with other pragmatic tasks seem to be related to the high frequency of working memory deficits. Corroborating this hypothesis, another study has shown involvement of working memory in narrative discourse without inference demand.^7^ Further, research on the inferential pragmatic component alone also shows a relationship with working memory when storage demand increases.^45^

It is important to note, however, that when compared to the RBD group, the TBI group had a greater percentage of EF deficits on different tasks combined with working memory and verbal fluency tasks. This finding suggests a more global dysexecutive syndrome in TBI population compared with RBD, possibly associated to more frequent compromise in pragmatic tasks. Moreover, in TBI cases only, two patients showed dissociation for presence of executive deficit (working memory), unaccompanied by pragmatic deficit or discursive impairment. These results suggest that pragmatic deficits may co-occur but cannot be completely explained by executive impairment. This research finding highlights the complex interaction between pragmatics, EF and memory, which represent real-life demands. Despite being highly correlated with each other, pragmatic and executive abilities can be dissociated.

No dissociations were found in the RBD group where all participants who presented pragmatic deficits also presented executive deficit, suggesting that pragmatic deficits co-occur with executive impairment and might be related. A study conducting a cluster analysis in RBD individuals found that pragmatic deficit can exist alone without executive impairment, suggesting that these deficits can co-occur, yet be unrelated, in some individuals.^40^ Similar results were discussed by Martin and McDonald,^37^ confirming an important contribution of EF in pragmatic processing, but this could not explain differences between RBD and controls. Nevertheless, the role of inhibition in the understanding of non-literal language was suggested by Champagne, Desautels and Joanette.^1^

Taken together, the findings in TBI and RBD patients raise challenging questions about the differential role of each cognitive component in pragmatics which warrant further investigation.

### Final comments

This study has some limitations that should be mentioned. First, all data discussed in this article was drawn from specific cases which in turn may not be representative of the TBI and RBD population as a whole. Second, discursive tasks entailed an overall assessment of several mnemonic, executive, linguistic and pragmatics components which cannot be individually analyzed. Consequently, results were biased by this associative demand, which on the other hand can be considered an accurate tool to simulate more ecological assessment of pragmatic language. In addition, the lesion sites of both groups may account for the findings,^10^ since while many RBD subjects could have middle central medial artery stroke, TBI patients tend to have more diffuse damage to the brain or greater frontal damage.^46^ In addition, the TBI group had a history of psychiatric disorders, a factor which may have contributed to the observed deficits. The association between psychiatric disorders and TBI is highly prevalent.^47,48^ Another limitation of the study is the relatively small sample size, which precluded inferential statistical analysis. Notwithstanding, case studies are very important in clinical neuropsychology.^49,50^ Future studies should investigate larger, more homogeneous samples with a greater number of ecological pragmatic and EF paradigms in order to identify pragmatic and executive dissociations and associations more closely related to daily complaints.
